# Physical activity and psychopathology: are long-term developmental trajectories of physical activity in children and adolescents associated with trajectories of general mental health problems and of attention-deficit hyperactivity (ADHD) symptoms?

**DOI:** 10.1007/s00787-023-02352-z

**Published:** 2024-02-12

**Authors:** Parisa Ganjeh, York Hagmayer, Thomas Meyer, Ronny Kuhnert, Ulrike Ravens-Sieberer, Nicole von Steinbuechel, Aribert Rothenberger, Andreas Becker

**Affiliations:** 1 Department of Child and Adolescent Psychiatry and Psychotherapy, University Medical Center Göttingen, Göttingen, Germany; 2 Department of Cognitive Science and Decision Psychology, Georg-Elias-Müller-Institute for Psychology, University of Göttingen, Göttingen, Germany; 3 Department of Psychosomatic Medicine and Psychotherapy, University Medical Center Göttingen, Göttingen, Germany; 4 Unit Mental Health, Department of Epidemiology and Health Monitoring, Robert Koch Institute, Berlin, Germany; 5 Department of Child and Adolescent Psychiatry, Psychotherapy and Psychosomatics, University Medical Center Hamburg-Eppendorf, Hamburg, Germany; 6 Institute of Medical Psychology and Medical Sociology, University Medical Center Göttingen, Göttingen, Germany

**Keywords:** Physical activity, General mental health problems, ADHD, Developmental trajectories, Children, Adolescents

## Abstract

A medium-to-high level of physical activity (PA) may have at least a short-term positive effect on psychopathology in children and adolescents. Hence, the objective of this study was to investigate the long-term effects of PA in non-adult age groups on their general mental health problems and/or ADHD symptoms, using trajectories of concurrent development over a period of 10 years. This study employed data from the German Health Interview and Examination Survey for Children and Adolescents (KiGGS) collected at three time points (baseline, Wave 1, Wave 2, over 10 years) from 17,640 children and adolescents. Using parent-reported data from the Strengths and Difficulties Questionnaire (SDQ), different developmental trajectories of general mental health problems (SDQ-total) and ADHD symptoms (SDQ-H/I) were identified with latent class mixed models (LCMM) statistics. This was also applied to parent- and self-reported data of three levels of PA. The latter was assessed according to WHO recommendations. The joint probability of class membership for SDQ-total as well as ADHD symptoms with PA was calculated to generate the concurrent developmental trajectories between variables. Results showed a 4-class trajectory model for both SDQ-total and ADHD symptoms among boys and girls. The majority of children and adolescents showed “low general difficulties” and “low ADHD symptoms” over the period of 10 years. Three distinct trajectories in boys and four distinct trajectories in girls were found for PA. Most of the participants showed an “increasing–decreasing activity” trajectory. No statistically significant correlations were found between the different SDQ-total or ADHD symptom trajectories and the trajectories of PA in the two genders. Taken together, our findings did not indicate any significant relationship between waxing and waning PA course over 10 years and various classes of mental health problems for children and adolescents. In contrast to our cross-sectional findings, no steady long-term medium/high-level of PA was present, which could (at least partly) explain the non-significant findings.

**Supplementary Information:**

The online version contains supplementary material available at 10.1007/s00787-023-02352-z.

## Introduction

Most mental health problems begin in childhood or adolescence, and 10–20% of children and adolescents are thought to have a diagnosable mental health condition [1]. Although various psychosocial and cognitive disorders can already occur in childhood and adolescence, the first symptoms of a psychiatric disorder such as schizophrenia usually appear later in adulthood [2]. Therefore, not only approaches for early detection but also for early prevention and low-level support are needed. In recent years, it has been shown that, according to WHO recommendations, high levels of weekly physical activity (PA) may be a helpful way to improve mental health problems in children and adolescents, at least in the short-term [3–13]. Because mental health problems in children and adolescents are often long-lasting (although they may wax and wane like anxious, depressive or obsessive–compulsive behavior), the question arises as to whether ongoing, regular moderate-to-high levels of PA can also be beneficial in these long-term cases. Looking at the association of concurrent long-term developmental trajectories of PA and mental health problems may be helpful in finding an answer. Considering that physical inactivity is a behavioral risk factor for mental illness [14], children and adolescents with consistently moderate-to-high levels of PA should be better off in the long run. But there still exists a gap of knowledge. Thus, our epidemiological approach (using data from a general child-to-adolescent population) tries to clarify whether PA may be helpful along the developmental course of psychopathology.

Developmental trajectories of mental health symptoms seem to differ widely among populations [15] and selected mental health issues [16–22]. Although children's developmental trajectories are reliable across different forms of mental health problems [17], several authors have highlighted categories of children and adolescents with increasing or decreasing symptoms [18, 23] using growth-mixture modeling techniques. For example, the Strengths and Difficulties Questionnaire (SDQ) subscale Emotional Symptoms showed four trajectories of depressive symptoms in a sample of 4983 Australian children: low-stable (75%), decreasing (11%), increasing (9%), and high and increasing (6%) groups [24]. Furthermore, in another large population of children aged 3–17 (*n* = 10,648), four different trajectories of conduct problems were reported: early-onset persistent, adolescence-onset, childhood-limited, and low level [25]. Hence, the diversity of trajectories of mental health problems in children and adolescents, as can also be seen in other studies [17, 26–28], makes it difficult to predict the long-term outcomes of these young study participants. Therefore, looking at long-term, concurrent trajectories may add knowledge and provide a better empirical basis concerning the development of symptoms in childhood.

Related to ADHD, cross-sectional and longitudinal follow-up studies have demonstrated that PA may have a positive impact on general mental health problems and ADHD symptoms [29, 30], although previous studies have indicated that there is considerable heterogeneity in the developmental course of ADHD symptoms [15, 31–41]. Furthermore, in 1571 children and adolescents (7–15 years old) the growth mixture model for hyperactivity/impulsivity found three classes, different for boys and girls [42]. This heterogeneity of study results presents a challenge for describing practically useful and reproducible developmental trajectories for ADHD symptoms.

Concerning PA, several studies have reported also different developmental trajectories for PA [43–50]. According to a recent systematic review of distinct trajectories of PA, three or four different trajectory classes were most frequently found among the reviewed studies, and several of the trajectories described a decline in PA during later childhood and adolescence [51]. Further, there is limited evidence on the concurrent trajectories of PA in daily life and mental health. More information on this issue could help to better understand the meaning of the probable association between these heterogeneous trajectories. Hence, the present epidemiological study examined the trajectories of general mental health problems, ADHD symptoms, and PA over a period of more than 10 years in a large representative sample. For each parameter we expected different classes of trajectories and assumed that the resulting trajectories of mental health problems and PA are related to each other.

## Methods

### Participants and procedures

**Fig. 1:**
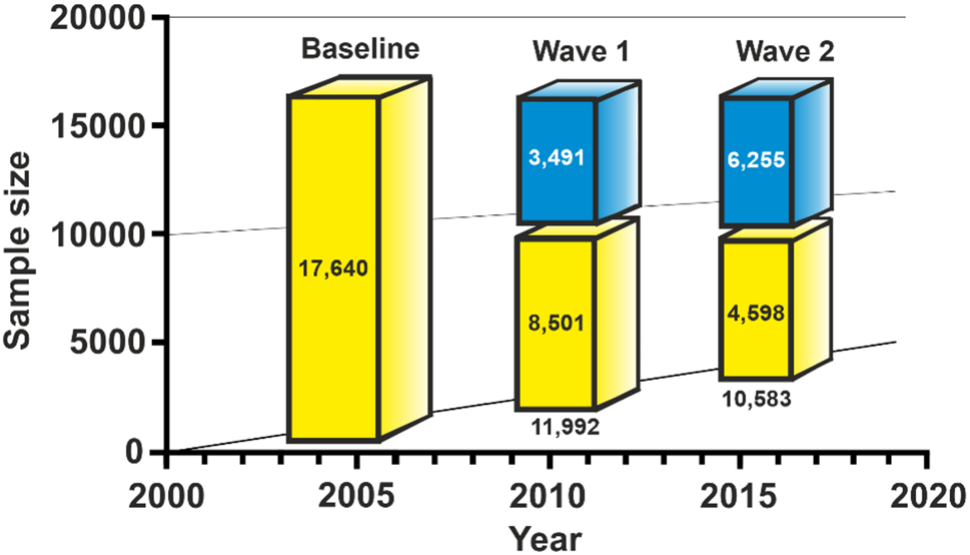
Longitudinal sample sizes of the KiGGS study. Yellow bars: longitudinal data up to 17 years old (the data used for analyses in the current study). Blue bars: longitudinal data at Wave 1 and Wave 2 older than 17 years

The longitudinal and large German Health Interview and Examination Survey for Children and Adolescents (KiGGS), which was carried out by the Robert Koch Institute, Berlin, served as the data source for this study. In other reports [52–54], the goal and design of the KiGGS study have been described in detail. Data were collected in three waves covering the periods from 2003 to 2006 (Baseline), 2009 to 2012 (Wave 1), and 2014 to 2017 (Wave 2). In total, 8656 girls and 8985 boys between the ages of 0 and 17 participated at baseline along with their parents. At 167 study locations dispersed throughout Germany, participants were chosen at random using a stratified sampling technique. For boys and girls, the response rates were 66% and 67%, respectively. After a lapse of roughly 6 years, the first follow-up survey (Wave 1) was conducted using a telephone questionnaire. To this end, 11,992 (68%) of the Baseline participants were again invited and took part in the examination for this wave [55]. In Wave 2, a cross-sectional and longitudinal sample of 15,023 children and adolescents aged between 0 and 17 years was used. There were 10,853 girls and boys with a response rate of 61.5% in the Wave 2 longitudinal sample [56, 57] (Fig. 1).

### Measurements

#### Physical activity (PA)

At baseline, PA was a composite variable created from a number of inquiries about the frequency with which children and adolescents participated in various sports and other physical activities. For children and adolescents aged 0–10 years, their parents or guardians provided data for PA. From the responses, three ordered categories of PA were calculated: 1 for low, 2 for medium, and 3 for high. For the assessments in Waves 1 and 2, the WHO’s recommendations for PA were taken into consideration [58]. The question “How many days is your child/are you at least 60 min physically active during a typical week?” was posed to both parents and adolescents. Respondents had seven options: from 0 for never to 7 for every weekday. Three categories of PA were also defined for the responses at Waves 1 and 2, namely: low = never to 2 days per week, medium = 3–5 days per week, and high = 6–7 days per week, to enable a comparison between time points. Consequently, PA was an ordered categorical variable with three levels for each of the three time points (low, medium, and high). The study protocol stated that because PA was evaluated by both parents (for children aged 0–10) and teenagers, it is a mixed-report variable. This choice was made since adolescents may more reliably provide behavioral information about themselves than younger children, due to their more developed memory and self-perception processes. Additionally, studies have shown that there is little agreement between parents’ and adolescents’ reports of PA [59–61], and that the agreements that do exist are typically related to organized PA [62]. Thus, the use of mixed reports of PA in this study was justified.

#### Mental health problems and ADHD symptoms

The Strength and Difficulties Questionnaire (SDQ) was used to assess mental health [63]. The SDQ consists of 25 items that ask respondents to rate statements on a scale of 0 (not true), 1 (somewhat true), and 2 (certainly true). The SDQ has five sub-scales: prosocial behavior, peer problems, hyperactivity/inattention, conduct problems, and emotional problems. Each subscale consists of five items (Prosocial Behavior: Items 1, 3, 5, 7, 9, Peer Problems: Items 2, 4, 6, 8, 10, Hyperactivity/Inattention: Items 11, 12, 13, 14, 15, Conduct Problems: Items 16, 17, 18, 19, 20, Emotional Problems: Items 21, 22, 23, 24, 25). The subscale scores range from 0 to 10, with higher scores indicating more difficulties, except for the Prosocial Behavior subscale, where higher scores indicate better behavior. Except for prosocial behavior, the total difficulties score is calculated by combining the scores of the subscales. The SDQ measures an individual’s emotional and behavioral well-being. Higher ratings on specific subscales may suggest potential problems. While there are no strict cut-offs for clinical diagnosis, scores exceeding specific thresholds may necessitate additional evaluation or intervention. For Total Difficulties, scores from 0 to 13 are generally considered within the normal range. Scores from 14 to 16 fall within the borderline range, suggesting some concerns, and scores of 17 and above may indicate a higher level of emotional and behavioral difficulties, potentially warranting further assessment or intervention. For individual sub-scales, scores from 0 to 2 are generally considered within the normal range. Scores from 3 to 4 are in the borderline range, indicating some concerns. If a subscale has a score of 4 or higher, it may suggest challenges in the specific area it covers, and additional evaluation or intervention may be advisable (e.g., emotional problems ≥ 5, conduct problems ≥ 4, hyperactivity/inattention ≥ 7, peer problems ≥ 4, prosocial behavior ≤ 4) [64]. In the current analysis, the total difficulties score and the hyperactivity/inattention score (SDQ-H/I) were used as indicators for general mental health problems and ADHD symptoms, respectively, and were taken from the parent-rated reports. The psychometric properties of the SDQ were examined in normal [65] and clinical [66] samples of German children and adolescents. It was demonstrated that the questionnaire is a reliable tool for identifying psychiatric patients [65, 66]. The SDQ-H/I sub-scale and the overall SDQ score in this study had Cronbach’s alphas of 0.80 and 0.76, respectively. It is also interesting to note that the scores of the Child Behavior Checklist [65] and the outcome scores of the SDQ have a significant correlation.

### Statistics

Descriptive statistics were conducted by IBM SPSS Statistics for Windows, Version 26.0 (International Business Machines Corporation, New York, USA). To identify different developmental trajectories, we carried out latent class mixed models (LCMM), using the R package lcmm [67]. First, we used latent class mixed models to group children into different classes of trajectories according to their development of general mental health problems (SDQ-total), ADHD symptoms (SDQ-H/I), and PA from baseline to Wave 2. In our models, shapes of trajectories were polynomial including a linear and quadratic trend over time. No random effects were included in the models. Models including one (basic) to five classes of trajectories were computed and compared. The Bayesian information criterion (BIC), the Akaike information criterion (AIC), entropy, and class size were used for comparisons [68]. The model with the lowest BIC and AIC and higher entropy values is the one that fits the data the best. We chose the model based on whether classes included a substantial number of participants and whether classes were of conceptual significance, i.e., showed clearly distinct trajectories that captured different developments over time. In order to investigate the relationship among trajectories of PA and mental health problems (SDQ-total) as well as trajectories of PA and symptoms of ADHD, we used crosstabs and calculated Cramer’s *v* as a measure of association and tested it for significance. Cramer’s *v* has ranges between 0 and + 1, and there is no association when the value is close to 0. A Cramer’s *v* value greater than 0.25 is defined as a very strong relationship.

## Results

### Characterization of the study sample

The total sample at Baseline was 17,640 children and adolescents (8654 girls and 8986 boys) aged 0–17 years. We used data for children and adolescents up to 17 years old because parent-reported SDQ data were only available up to this age. The exact numbers and descriptive characteristics of the longitudinal sample at Baseline, Wave 1, and Wave 2 are shown in Table 1. Note that the number of participants declines across the three waves due to participants’ dropping out.

**Table 1:** Descriptive characteristics of the study population at Baseline, Wave 1, and Wave 2

	Baseline				Wave 1				Wave 2			
	Boys (*n* = 8986)		Girls (*n* = 8654)		Boys (*n* = 4274)		Girls (*n* = 4274)		Boys (*n* = 2258)		Girls (*n* = 2340)	
	Mean	SD	Mean	SD	Mean	SD	Mean	SD	Mean	SD	Mean	SD
Age (years)	8.54	5.053	8.48	5.09	11.77	3.37	11.70	3.39	14.01	1.99	13.98	1.98
SDQ-emotion	1.69	1.79	1.87	1.80	1.75	1.75	2.15	1.91	1.45	1.73	1.94	1.94
SDQ-behavioral problems	2.09	1.60	1.78	1.44	2.09	1.58	1.79	1.44	1.69	1.50	1.39	1.33
SDQ-hyperactivity inattention	3.54	2.34	2.72	2.31	3.32	2.25	2.55	2.05	2.99	2.15	2.17	1.89
SDQ-peer problems	1.56	1.68	1.32	1.53	1.42	1.57	1.17	1.40	1.50	1.73	1.15	1.43
SDQ-prosocial behavior	7.55	1.77	8.06	1.62	8.08	1.62	8.62	1.41	7.71	1.82	8.29	1.59
SDQ-total	8.88	5.34	7.69	4.86	8.58	5.09	7.66	4.78	7.63	5.06	6.65	4.69
Physical activity												
Low	2668		3337		646		1048		440		774	
Medium	2833		2589		2090		2015		1081		1092	
High	2992		2229		1340		1011		607		373	
Total	8493		8155		4076		4074		2128		2239	

### Latent trajectories of general mental health problems and ADHD symptoms

Unconditional latent class mixed models (LCMM) with 1–5 latent trajectory classes were estimated separately for general mental health problems (SDQ-total) and ADHD symptoms in boys and girls. For more details on the fit indices of the models and group sizes of classes, see Appendix A (Tables A1–A4).

**Fig. 2:**
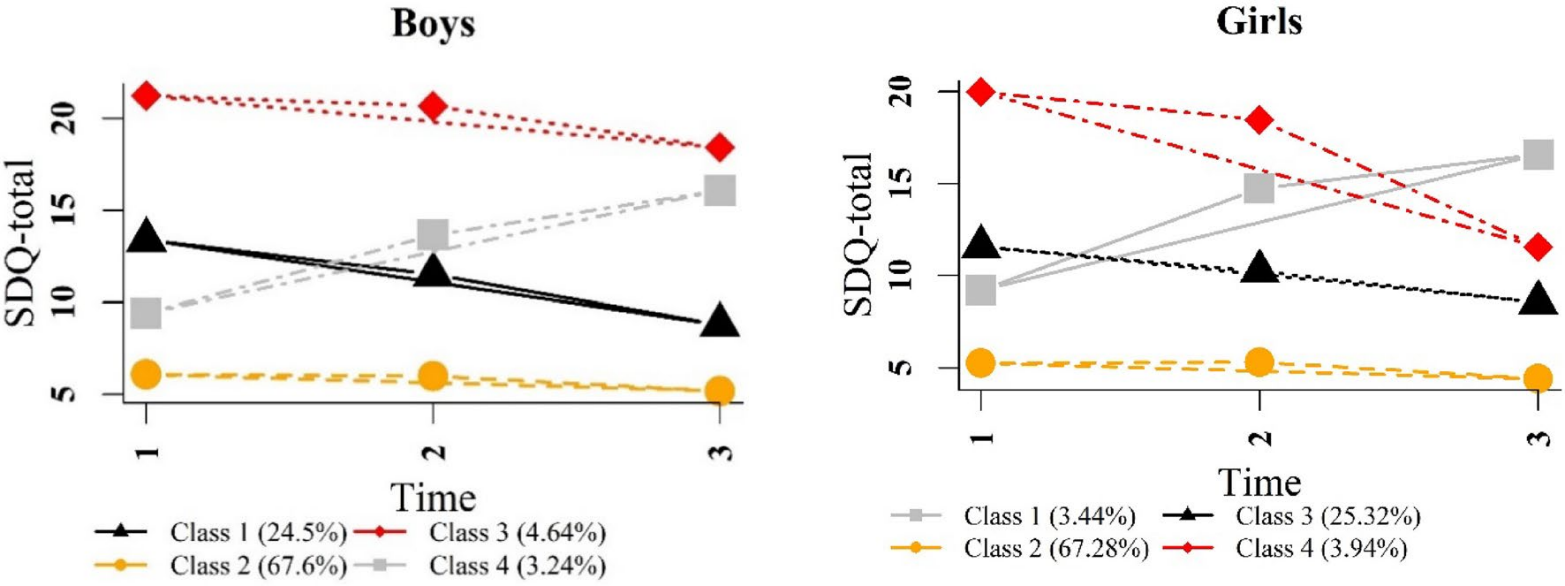
SDQ-total trajectories for boys and girls in four-class models

**Fig. 3:**
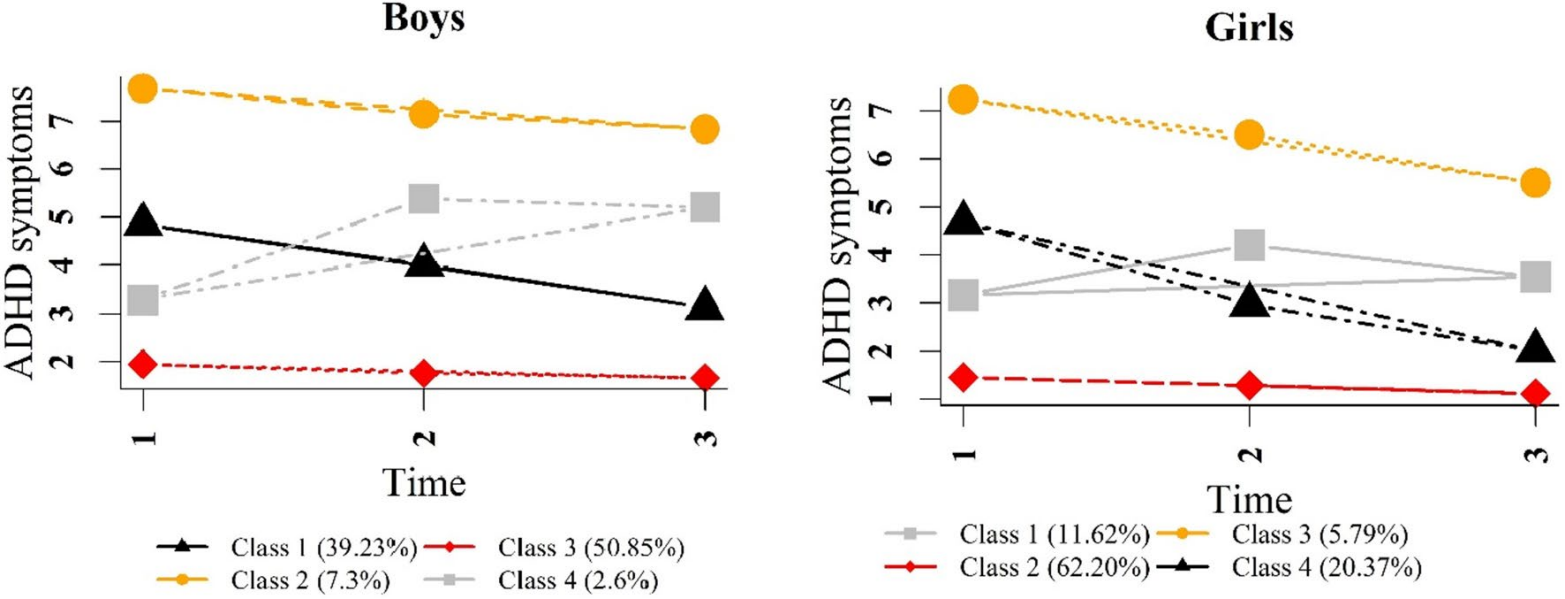
ADHD symptom trajectories for boys and girls in the four-class models

**Fig. 4:**
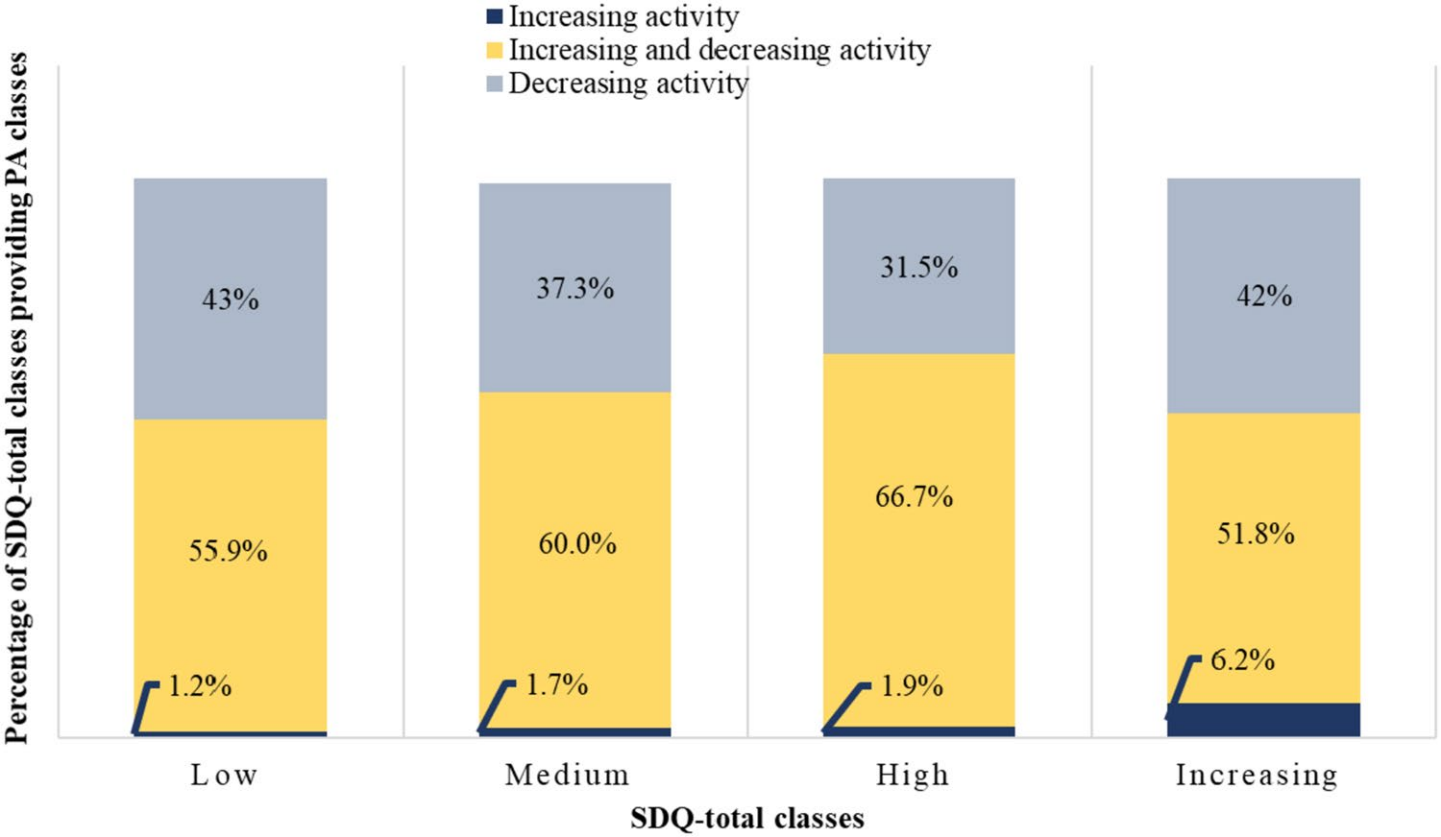
Association between SDQ-total and PA classes in boys (*χ*^2^(6) = 0.10219, *p* = 1; Cramer’s *v* = 0.113)

After comparing the different models, the 4-class model was chosen for SDQ-total and ADHD symptoms in both boys and girls. This model type includes a specific group with increasing trajectories in SDQ-total and ADHD symptoms for boys and girls (i.e. mental health problems increased over time, see Figs. 2 and 3). We did not choose the five-class model, since it only had a slightly better fit than the four-class model, but groups sizes of classes decreased and some of the models had lower entropy than the corresponding four-class models. Figures 3 and 4 show the classes of trajectories identified by the four-class models for both genders.

For the SDQ-total score, the largest class (depicted in yellow in Fig. 2) included 67.6% of boys and 67.3% of girls and was labeled as “low difficulties”, indicating that these class members had low mental health problems at every time point. The second biggest class (depicted in black in Fig. 2) labeled as “medium difficulties” had a decreasing trend and encompassed 24.5% of boys and 25.3% of girls, respectively. The third class started with a high initial score of SDQ-total, which decreased over time (depicted in red in Fig. 2), and was therefore labeled “high difficulties”. It included 4.6% and 3.9% of boys and girls. The smallest class (depicted in gray in Fig. 2) showed an increasing trend and was therefore named “increasing difficulties”. It included 3.2% of boys and 3.4% girls.

Four distinct classes of trajectories were identified for ADHD symptoms. The majority of boys (50.9%) and girls (62.2%) were in the class entitled “low symptoms” (depicted in red in Fig. 3). All members of this class had no or low ADHD symptoms at all three time points. The second largest class (depicted in black in Fig. 3) included 39.2% of boys and 20.4% of girls and started with medium scores of ADHD with a decreasing trend labeled “medium symptoms”. The class named “high symptoms” (depicted in yellow in Fig. 3) included 7.3% of boys and with 5.8% the lowest percentage of girls. Children and adolescents in this class began with high ADHD symptoms at baseline and showed a decreasing trend to Wave 2. Finally, the class labeled “increasing symptoms” (depicted in gray in Fig. 3) encompassed 11.6% of girls and the lowest percentage of boys (2.6%). ADHD symptoms in this class increased from baseline to Wave 1 and then decreased slightly to Wave 2 for girls, whereas they remained relatively stable for boys.

### Latent trajectories of PA

To determine the number of trajectory classes for PA, the literature was reviewed. Three or four classes of trajectories were most frequently reported for children and adolescents according to a systematic review of various trajectories of PA by Lounassalo et al. [51]. Therefore, we only fitted models with three and four classes to the data for PA (for more details see Appendix B, Tables and Figures B1-B2). For boys, the 3-class approach was selected due to very few members in the fourth class (less than 1%). Class number 1 was named “increasing activity” with very few participants (1.4%). The second class included most boys (58.3%) and was named “increasing and decreasing activity”. In this class, PA increased from baseline to Wave 1, but decreased after that. Class number 3, named “decreasing activity”, included 40.3% of boys. The trajectory of PA decreased in this class. Table 2 shows the development of the level of PA at the three time points for all classes.

**Table 2:** Distribution of PA levels at three time points for the three classes of trajectories identified in boys

	Baseline (Time 1)			Wave 1 (Time 2)			Wave 2 (Time 3)		
	Low (%)	Medium (%)	High (%)	Low (%)	Medium (%)	High (%)	Low (%)	Medium (%)	High (%)
Class 1 (increasing activity)	100	0	0	0	0	100	0	0	100
Class 2 (increasing and decreasing activity)	52	46	0.0	24	61	15	33	60	7
Class 3 (decreasing activity)	0	13	87	8	44	47	14	49	37

For girls, a 4-class model was chosen, because the model with three classes did not converge. The first class contained girls (1.4%), who began their PA at low and medium levels and subsequently decreased their activity. The label given to this class type was “low and decreasing activity”. Class number 2, which included the highest percentage of girls (71.5%), was named “increasing and decreasing activity”. The third class included the lowest number of girls (0.7%) and was labeled as “increasing activity”. Class number 4 was named “high and decreasing activity”. This class included 26.5% of girls who displayed a decreasing trend in PA starting from a high level. The development of the PA levels at the three time points for the four classes are depicted in Table 3.

**Table 3:** Distribution of levels of PA at three time points for the four classes of trajectories identified in girls

	Baseline (Time 1)			Wave 1 (Time 2)			Wave 2 (Time 3)		
	Low (%)	Medium (%)	High (%)	Low (%)	Medium (%)	High (%)	Low (%)	Medium (%)	High (%)
Class 1 (low and decreasing activity)	47	53	0	100	0	0	100	0	0
Class 2 (increasing and decreasing activity)	55	45	0	24	54	22	33	55	13
Class 3 (increasing activity)	100	0	0	0	0	100	0	0	100
Class 4 (high and decreasing activity)	0	0	100	25	50	26	31	50	18

### Relationship of classes of trajectories of general mental health problems (SDQ-total) and PA

**Fig. 5:**
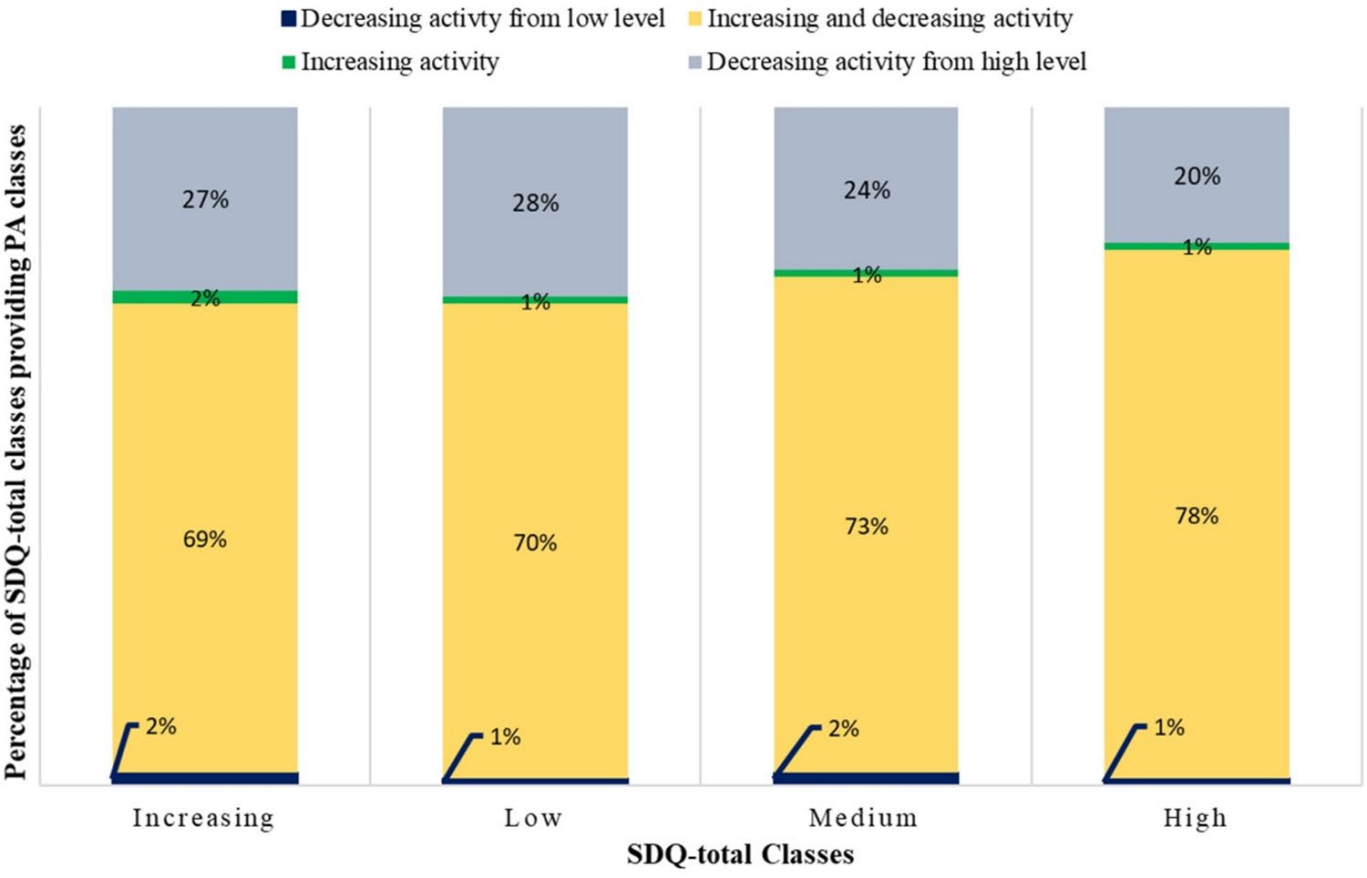
Association between SDQ-total and PA classes in girls (*χ*^2^(9) = 0.04319, *p* = 1; Cramer’s *v* = 0.06)

The relationship between trajectories of SDQ-total and PA is depicted in Fig. 4 for boys and in Fig. 5 for girls. These figures show that the distribution of classes of PA was very similar for the different classes of SDQ-total for boys and girls. This indicated no relation among classes of PA and SDQ trajectories. The statistical analysis found only small, non-significant associations between classes of SDQ-total and PA trajectories for boys (*χ*^2^(6) = 0.10219, *p* = 1; Cramer’s *v* = 0.113) and girls (*χ*^2^(9) = 0.04319, *p* = 1; Cramer’s *v* = 0.06).

### Relationship of classes of trajectories of ADHD symptoms and PA

**Fig. 6:**
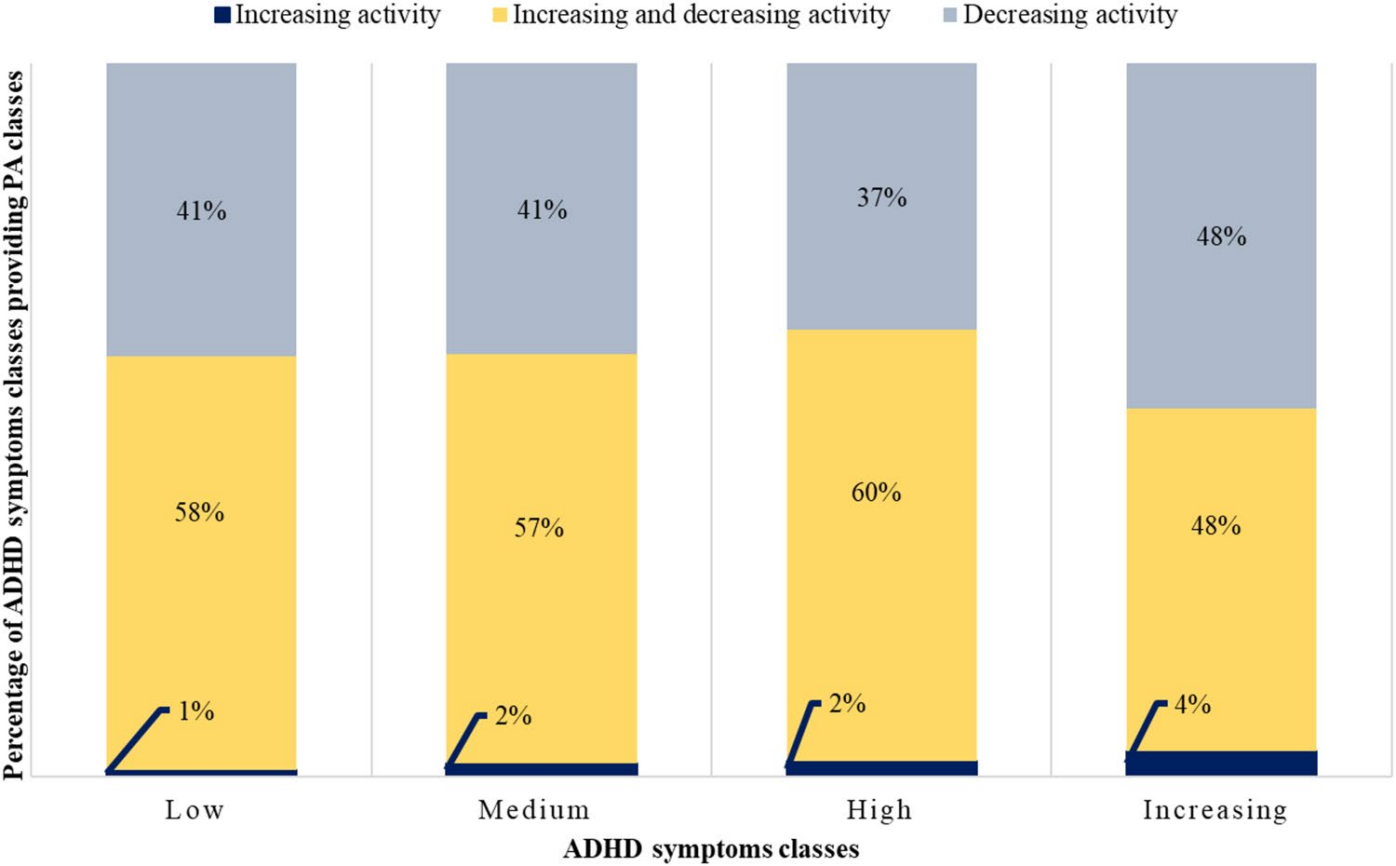
Association between ADHD symptoms and PA classes in boys (*χ*^2^(6) = 0.04781, *p* = 1; Cramer’s *v* = 0.077)

**Fig. 7:**
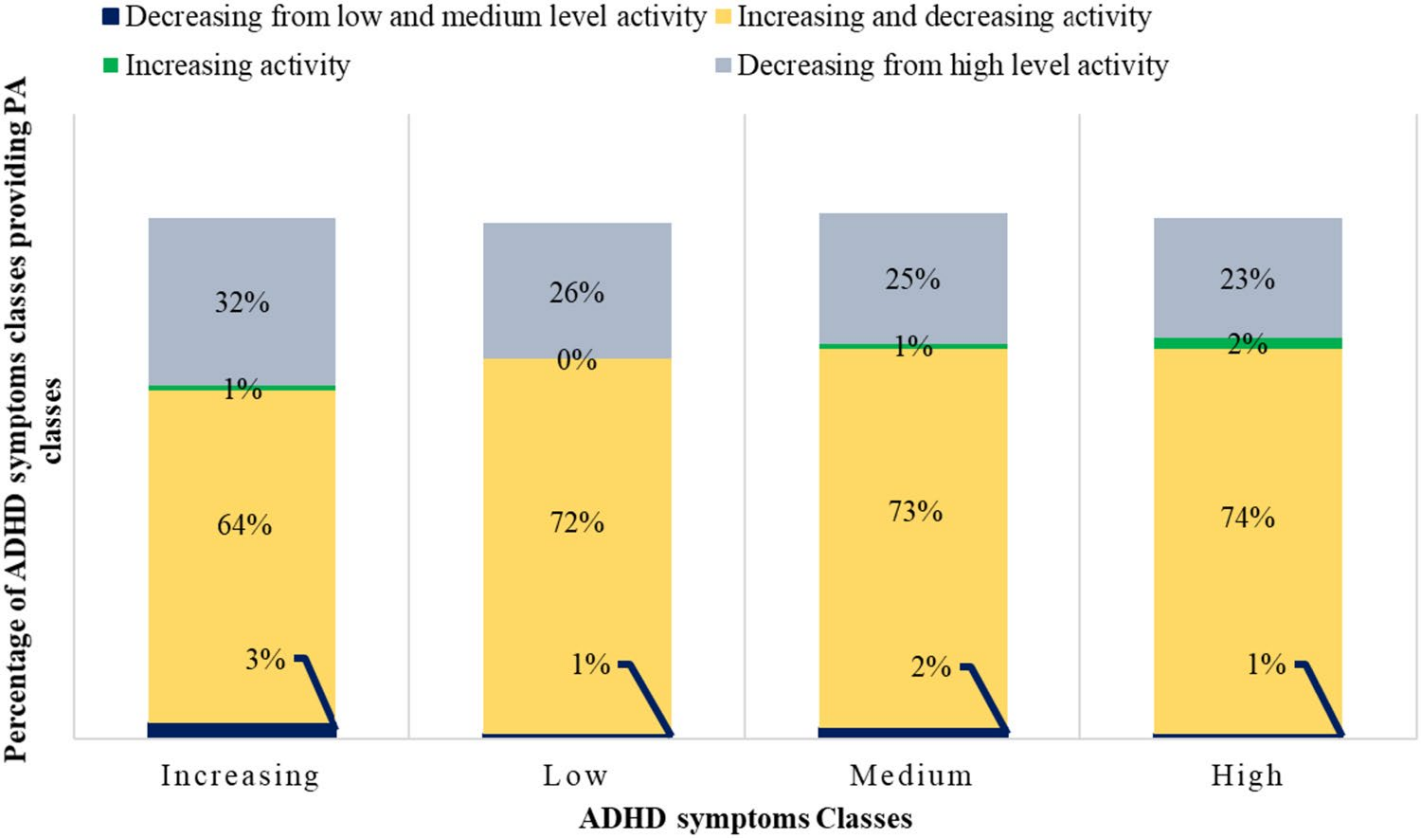
Association of ADHD symptoms and PA classes for girls (*χ*^2^(9) = 0.0511, *p* = 1; Cramer’s *v* = 0.065)

We used the same graphs to depict the relationship of classes of trajectories of ADHD symptoms and classes of trajectories of PA. Figure 6 shows the results for boys and Fig. 7 for girls. Again, the distribution of classes of PA across the classes of ADHD symptoms looked rather similar, indicating no relation. The classes of trajectories were not significantly associated for boys (*χ*^2^(6) = 0.04781, *p* = 1; Cramer’s *v* = 0.077) and girls (*χ*^2^(9) = 0.0511, *p* = 1; Cramer’s *v* = 0.065).

## Discussion

There exists some indication of an overall positive effect of PA on mental health problems in children and adolescence [5–7, 9–11]. However, it remains unclear whether this is also the case when looking at different long-term developmental trajectories of psychological problems from childhood to late adolescence. The present study investigated the associations between PA trajectories and those of general mental health problems and ADHD symptoms in an epidemiological cohort of German children and adolescents over a period of 10 years. Here we identified four different trajectories of general mental health problems and ADHD symptoms as well as a 3-class trajectory approximation for PA in male study participants and a 4-class model in female participants. However, no statistically significant associations could be found between the different trajectories of general mental health problems/ADHD symptoms and optimal classes of PA, which holds for both genders. Unexpectedly, there was also no consistent long-term group of PAs at the medium to high level.

The results from our trajectory analysis revealed that models with four different classes for trajectories of general mental health problems (SDQ-total) as well as ADHD symptoms fit best for the data. The classes for both boys and girls in relation to the SDQ-total score were labeled as “low difficulties”, “medium difficulties”, “high difficulties”, and “increasing difficulties”. These findings were consistent with previous work which showed developmental heterogeneity in different kinds of mental health problems for children and adolescents [18, 19, 27, 28]. They are also in line with studies that grouped most of their population into low mental health problems [17, 24]. Approximately two-thirds of the boys and girls (67%) in our cohort were classified as having low difficulties. Although some studies reported gender-specific trajectories for boys and girls [17, 24, 27], we found similar trajectories for general mental health problems for both genders, as was also shown in some previous studies [21]. According to our findings, there was only a difference in the slope of declining symptoms between boys and girls in the high difficulties class. Girls experienced a steeper decline of mental health problems than boys (from baseline to Wave 2). This may reflect, at least partially, that girls are more likely to seek help than boys. The second large trajectory in our findings included boys and girls with initial medium difficulties that decreased over the follow-up period of 10 years. This was observed not only from preschool age to pre-adolescence, but a decreasing trend was also seen from pre-adolescence to adolescence. These observations are in contrast with findings by Parkes et al. [21] demonstrating a medium increasing trajectory among children from preschool to about 8 years of age. The different age groups and the use of the internalizing subscale of the SDQ in the Parkes et al. study may explain the differences.

Most of the boys and girls showed low symptoms of ADHD and even some improvement over the three time points. Similar findings were reported in earlier work [39, 41]. In line with the age-related decline in ADHD symptoms [31, 69, 70], two classes of ADHD symptom trajectories (medium and high symptoms) were identified among boys and girls in the current study, indicating decreasing trajectories for children and adolescents with high and medium scores of ADHD symptoms. However, the high symptom trajectory (after a decline) showed a high score of ADHD symptoms, especially for boys. A smaller percentage of boys (2.6%) and girls (11.6%) had an increasing pattern of ADHD symptoms. Over three time points, the linear lines showed a rising trend for both genders. However, the quadratic lines demonstrated that the level of ADHD symptoms increased from baseline to Wave 1 with a steeper slope for boys. Then, the trend from the preadolescent age (Wave 1) to adolescence (Wave 2) was stable for boys while slightly declining for girls. These findings differ to some extent from earlier work by Murray et al., who showed that symptoms of ADHD as measured by the SDQ-H/I subscale increased to clinically significant levels by the time the age of 14 years was reached [39]. However, this study did not report results separately for boys and girls.

Although several gender differences could be detected, the developmental differences for psychopathological signs in the investigated age range seem to be rather small. The presented data showed a 3-class model for PA in boys and a 4-class model in girls. If this reflects a greater diversity in girls, remains an open question. Most of the boys and girls were categorized as belonging to the increasing and decreasing category of PA. The latter fits with reports that PA declines during the transitional period from childhood to adolescence [71]. Additionally, the second large class in both boys and girls was the class, where individuals experienced a decreasing trajectory after beginning with a high level of PA. The results were consistent with earlier studies that demonstrated a general decline in PA among children and adolescents [72, 73]. In addition to adolescent development changes, the decline in PA may be caused by a changing lifestyle (i.e. more sedentary behavior), altered transportation patterns, effects of industrialization, and an increased use of technology including tablets, mobile devices, and computers [74].

Contrary to expectations, we could not find a significant association between long-term trajectories of general mental health problems or ADHD symptoms on the one hand and PA trajectories on the other. In two earlier cross-sectional and longitudinal follow-up studies [29, 30], it was shown that high weekly PA may play a protective role concerning general mental health problems and ADHD symptoms. Unfortunately, in our trajectory study there was no trajectory group with a continuously high-level of PA; instead, PA trended to wax and wane. This fact might have contributed to a loss of the basically positive effect of PA on mental health problems. Probably, continuously practicing PA could be beneficial for developmental mental health.

## Strengths and limitations

One of the strengths of the current longitudinal study is the use of a reasonably large population sample size across a wide age range—from preschoolers to adolescents—at various time points to determine the trajectories of variables in a longitudinal approach. Another strength is that it examines the associations between different developmental trajectories (i.e. general mental health problems, ADHD symptoms) with PA. There are also some limitations. First, PA was measured with a single question, which produced a categorical variable. Hence, we could not use conventional joint trajectory methods to look at the concurrent trajectories between a categorical variable (PA) and continuous variables like SDQ-total or ADHD symptoms. Nevertheless, an adequate statistical approximation was applied. The second study limitation was the low entropy values of the models, which ranged from 0.53 to 0.68. Since there is no clear cut-off value for entropy, we considered other indicators before selecting a model [75]. The third limitation is that the study used only data from parent-reported questionnaires to determine general mental health and ADHD symptoms. Additionally, we did not include weighted factors in the analyses, which means that our results are not entirely representative of the German population. Further work is recommended on concurrent trajectories using multiple informants for developmental psychopathology and objective methods (e.g., accelerometer) to register PA data.

## Conclusion

In summary, this paper highlights the heterogeneity of developmental trajectories for general mental health problems, ADHD symptoms and PA in children and adolescents of both genders. The non-significant associations between long-term concurrent trajectories of general mental health problems/ADHD symptoms and PA, combined with our positive cross-sectional and follow-up results, may provide further evidence that developmental psychopathological features should be considered not only in a cross-sectional manner but also in terms of long-term developmental changes to better tailor diagnostic and treatment strategies to the individual.

## Supplementary Information

Below is the link to the electronic supplementary material.

Supplementary file1 (DOCX 186 KB)

## Data Availability

The datasets analyzed during the current study are available from the corresponding author on reasonable request.
