# Supplementary material for: Physical activity and psychopathology: are long-term developmental trajectories of physical activity in children and adolescents associated with trajectories of general mental health problems and of attention-deficit hyperactivity (ADHD) symptoms?
Source: Eur Child Adolesc Psychiatry. 2024 Feb 12;33(9):3067–78. doi: 10.1007/s00787-023-02352-z (PMC11424711; doi:10.1007/s00787-023-02352-z)
Supplement: Supplementary file 1 — Supplementary file1 (DOCX 186 KB) [file 787_2023_2352_MOESM1_ESM.docx]

**Appendix A. Model fit indices of LCMM with group size of classes for SDQ-total and ADHD symptoms in boys and girls**

Table A1. Model fit indices to identify trajectory classes of SDQ-total in boys

|  | LogLik | BIC | AIC | Entropy | Class 1 | Class 2 | Class 3 | Class 4 | Class 5 |
| --- | --- | --- | --- | --- | --- | --- | --- | --- | --- |
| Class 1 | -42457.84 | 84951.88 | 84923.68 | 1.00 | 100.00 |  |  |  |  |
| Class 2 | -41284.77 | 82641.94 | 82585.54 | 0.75619 | 16.6490 | 83.3509 |  |  |  |
| Class 3 | -40965.42 | 82039.44 | 81954.84 | 0.6920 | 28.3667 | 5.7649 | 65.8682 |  |  |
| **Class 4** | **-40859.99** | **81864.77** | **81751.97** | **0.6700** | **24.5039** | **67.6059** | **4.6495** | **3.2405** |  |
| Class 5 | -40775.91 | 81732.82 | 81591.83 | 0.6581 | 1.8081 | 63.6022 | 26.4412 | 4.2503 | 3.8980 |

Table A2. Model fit indices to identify trajectory classes of SDQ-total in girls

|  | LogLik | BIC | AIC | Entropy | Class 1 | Class 2 | Class 3 | Class 4 | Class 5 |
| --- | --- | --- | --- | --- | --- | --- | --- | --- | --- |
| Class 1 | -40654.70 | 81345.48 | 81317.40 | 1.00 | 100.00 |  |  |  |  |
| Class 2 | -39557.28 | 79186.72 | 79130.56 | 0.7476 | 82.6639 | 17.3360 |  |  |  |
| Class 3 | -39257.68 | 78623.60 | 78539.36 | 0.7173 | 23.8688 | 72.1025 | 4.0285 |  |  |
| **Class 4** | **-39124.53** | **78393.37** | **78281.06** | **0.6878** | **3.4478** | **67.2876** | **25.3205** | **3.9438** |  |
| Class 5 | -39037.69 | 78255.77 | 78115.37 | 0.6296 | 3.6051 | 2.4921 | 24.0987 | 60.5008 | 9.3031 |

Table A3. Model fit indices to identify trajectory classes of ADHD symptoms in boys

|  | LogLik | BIC | AIC | Entropy | Class 1 | Class 2 | Class 3 | Class 4 | Class 5 |
| --- | --- | --- | --- | --- | --- | --- | --- | --- | --- |
| Class 1 | -31067.13 | 62170.47 | 62142.26 | 1.00 | 100.00 |  |  |  |  |
| Class 2 | -30176.66 | 60425.73 | 60369.32 | 0.6086 | 23.9178 | 76.0821 |  |  |  |
| Class 3 | -29914.28 | 59937.17 | 59852.56 | 0.5936 | 40.7390 | 51.1319 | 8.12903 |  |  |
| **Class 4** | **-29866.68** | **59878.17** | **59765.36** | **0.5778** | **39.2375** | **7.30791** | **50.8504** | **2.60410** |  |
| Class 5 | -29793.93 | 59768.87 | 59627.86 | 0.5572 | 5.1378 | 47.8944 | 2.4398 | 35.7653 | 8.7624 |

Table A4. Model fit indices to identify trajectory classes of ADHD symptoms in girls

|  | LogLik | BIC | AIC | Entropy | Class 1 | Class 2 | Class 3 | Class 4 | Class 5 |
| --- | --- | --- | --- | --- | --- | --- | --- | --- | --- |
| Class 1 | -29217.33 | 58470.74 | 58442.65 | 1.00 | 100.00 |  |  |  |  |
| Class 2 | -28216.56 | 56505.30 | 56449.13 | 0.6942 | 78.4730 | 21.5269 |  |  |  |
| Class 3 | -27960.12 | 56028.49 | 55944.23 | 0.6604 | 31.2515 | 5.7743 | 62.9741 |  |  |
| **Class 4** | **-27879.83** | **55904.00** | **55791.66** | **0.5826** | **11.6211** | **62.2010** | **5.7985** | **20.3793** |  |
| Class 5 | -27813.29 | 55807.00 | 55666.58 | 0.5899 | 2.4039 | 20.2826 | 50.2899 | 4.6146 | 22.4087 |

**Appendix B. The fit indices and group size of classes for LCMM model of PA in boys and girls**

Table B1. Model fit indices to identify trajectory classes of PA in boys

|  | LogLik | BIC | AIC | Entropy | Class 1 | Class 2 | Class 3 | Class 4 |
| --- | --- | --- | --- | --- | --- | --- | --- | --- |
| **Class 3** | **-15544.22** | **31197.38** | **31112.45** | **0.5314** | **1.4279** | **58.3162** | **40.255** |  |
| Class 4 | -15521.05 | 31187.33 | 31074.09 | 0.5403 | 0.1941 | 65.5471 | 27.4274 | 6.8311 |

Figure B1. PA trajectories for boys for the three-class model

**
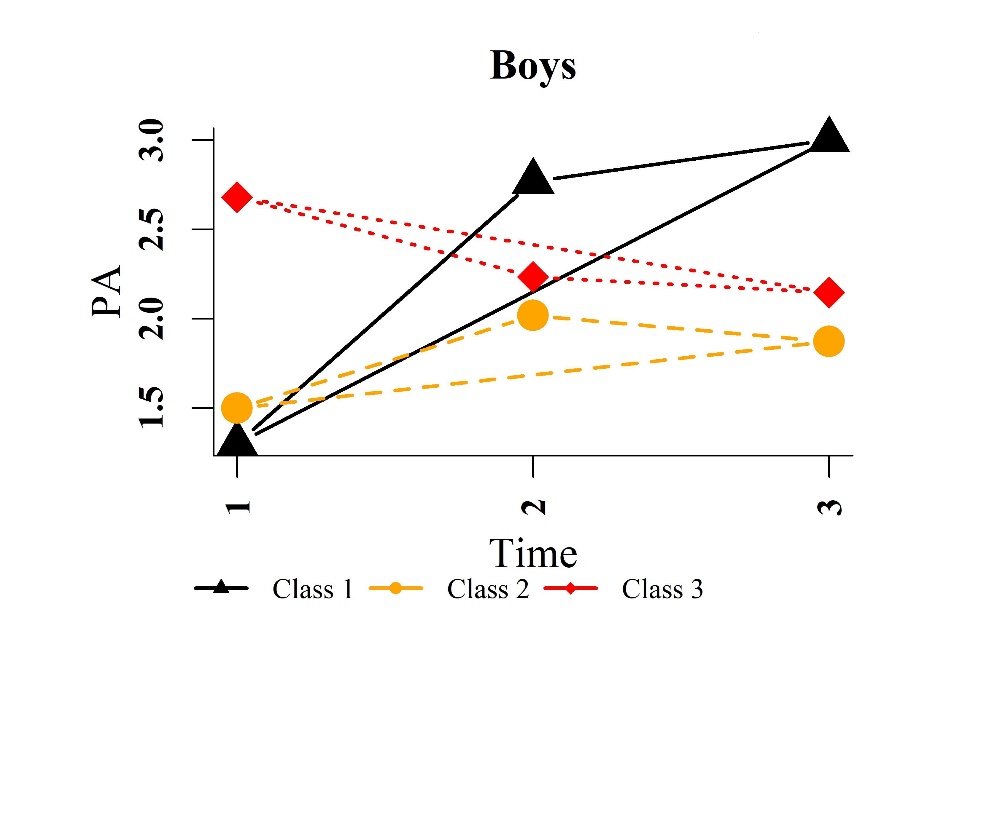
**

Table B 2. Model fit indices to identify trajectory classes of PA in Girls

|  | LogLik | BIC | AIC | Entropy | Class 1 | Class 2 | Class 3 | Class 4 |
| --- | --- | --- | --- | --- | --- | --- | --- | --- |
| **Class 4** | **-15330.32** | **30805.25** | **30692.63** | **0.6082** | **1.3773** | **71.4913** | **0.6649** | **26.4664** |

Figure B1. PA trajectories for girls for the four-class model

**
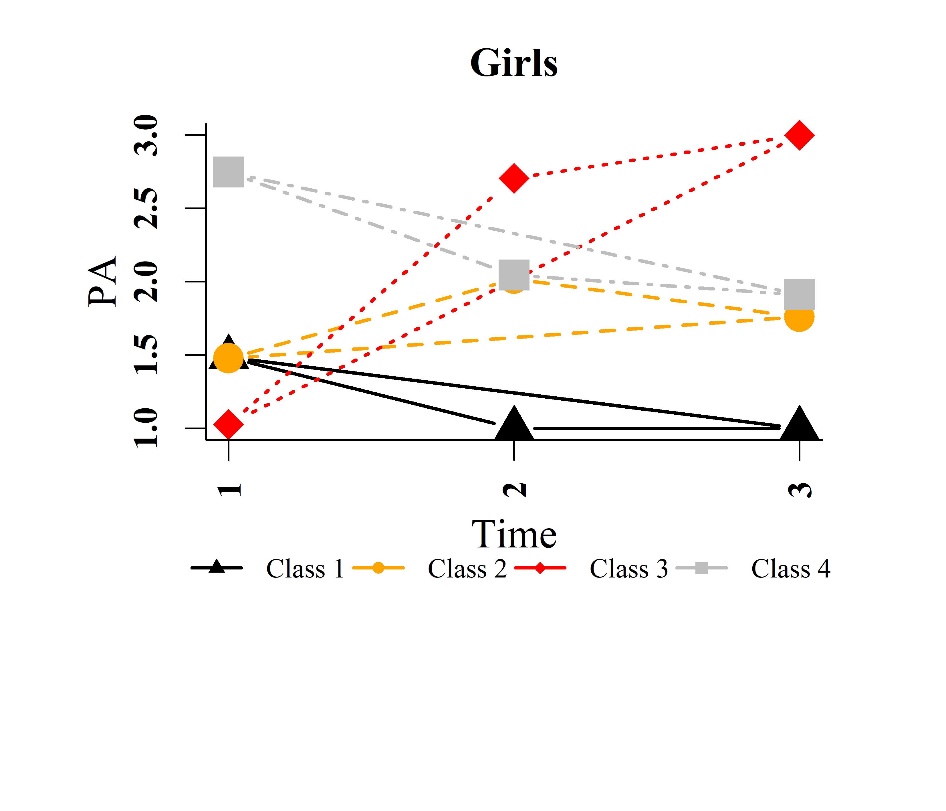
**
